# Memory accuracy, suggestibility and credibility in investigative interviews with native and non-native eyewitnesses

**DOI:** 10.3389/fpsyg.2023.1240822

**Published:** 2023-08-24

**Authors:** Arman Raver, Torun Lindholm, Philip U. Gustafsson, Charlotte Alm

**Affiliations:** Department of Psychology, Faculty of Social Sciences, Stockholm University, Stockholm, Sweden

**Keywords:** investigative interview, confidence-accuracy relationship, memory accuracy, suggestibility, credibility judgment, language barrier, non-native speaker

## Abstract

Legal practitioners sometimes obtain eyewitness testimonies from non-native language speakers, yet few studies examine the effects of language in investigative interviews. Here, we investigate how testifying in a non-native vs. native language affects memory accuracy, susceptibility to suggestions, and witnesses perceived credibility. After viewing a mock-crime film, participants in Study 1 (*N* = 121) testified through (1) free recall, (2) cued recall and (3) the Gudjonsson Suggestibility Scales adapted to the crime scenario used in the present study either in their native (Swedish) or a non-native (English) language. They also rated their confidence in their memories, self-perceived credibility and cognitive effort. Native and non-native witnesses did not differ in memory accuracy, susceptibility to suggestions, self-rated credibility or cognitive effort. Non-native (vs. native) speakers did however report lower confidence in their memories. In Study 2, another group of participants (*N* = 202) were presented with the testimonies from Study 1, and judged witnesses’ credibility. Non-native witnesses were judged as less credible than native speakers. Thus, while the lower confidence exhibited by non-native eyewitnesses did not correspond to their actual memory accuracy, it influenced observers’ judgments of their performance. The results provide important knowledge for legal practices when evaluating the reliability of testimonies from non-native vs. native speaking eyewitnesses.

## Introduction

Reporting a criminal event as an eyewitness in an investigative interview is often demanding. Not only would reporting about a criminal event lead to emotional discomfort, but the witness must also exert cognitive effort to retrieve information about the crime from memory, and further still, communicate this information in a comprehensible way. While it is cognitively taxing for any witness to report a criminal event (Hanway et al., 2021), testifying in a non-native, as opposed to a native, language is likely to require additional effort (Green, 1998; Hernandez and Meschyan, 2006; Abutalebi, 2008). One reason for this may be that interviewees in such cases must inhibit their native language since it competes with retrieval in the non-native language. This formed our rationale for conducting this research, as such language differences could potentially affect witnesses’ memory accuracy, confidence and suggestibility, and importantly, subsequent credibility judgments by independent judges. Despite these possible difficulties in non-native eyewitness reports, there is limited research examining how eyewitnesses report in a non-native vs. native language may affect the quality of witnesses’ memories in investigative interviews, and how this in turn may influence evaluators’ credibility judgments. The current study addresses these issues by examining how testifying in a native or non-native language affects witnesses’ actual accuracy, suggestibility and confidence, and how the quality of the testimonies of these witnesses are evaluated by observers.

### Language barriers to memory accuracy in investigative interviews

Witnessing a crime is typically unexpected, and often happens under conditions that are not optimal for remembering the event (Tulving and Bower, 1974; Albright, 2017). Witnessing a crime in a country where one does not speak the language can be extra taxing, and testifying in a non-native language can pose an additional challenge (Itzhak et al., 2017). While interpreters may be used in these situations, the police-interpreter-eyewitness interaction is not flawless. There is a plethora of possible risks, such as distortions in the translation of statements, which may affect the validity of the testimony (Lai and Mulayim, 2014; Dhami et al., 2017). Lack of resources, heavy workload, and logistical challenges sometimes also delay or hinder the use of an interpreter (Goodman-Delahunty et al., 2020). A few studies have investigated how reporting a crime event in a non-native language affects memory accuracy and suggestibility. For example, Alm et al. (2019) had native Swedish speakers report their memories of a mock-crime event either in their native language or in a non-native language (i.e., English). They found that witnesses reporting the event in a non-native language more often yielded to suggestive questions, and rated their own credibility as lower compared to native-language witnesses. Similarly, Hu and Naka (2022) investigated how reporting a crime event in witnesses’ native or a non-native tongue affected accuracy and the quantity of reported details of different types of information (agent, place, object, and action descriptions). Using a within-subjects design with bilinguals, they found that memories for some details (e.g., objects and actions) were more accurate when witnesses testified in their native tongue, while other details (place descriptions) were remembered better in the non-native language. Surprisingly however, participants conveyed more inaccurate information across all information categories when speaking in their native (vs. a non-native) tongue. Finally, Ernberg and Mac Giolla (2022) compared different means of conducting investigative interviews with crime victims with language barriers. Interviews were conducted either face-to-face with non-native speakers, face-to-face with native speakers through an interpreter, or by letting victims use the Self-Administered Interview in their native tongue (SAI; Hope et al., 2011). They found that the native-speaking participants (with either an interpreter or using the SAI) reported slightly more accurate details than the non-native speaking participants, although the differences were not statistically significant. In terms of proportion of accurate details overall, the non-native speaking participants were instead slightly more accurate than the native-speaking participants (in both conditions). In sum, these studies show inconsistent results, making it difficult to draw firm conclusions regarding the effects of language on memory accuracy.

### Suggestibility

Suggestibility refers to “the extent to which, within a closed social interaction, people come to accept messages communicated during formal questioning, as the result of which their subsequent behavioral response is affected” (Gudjonsson and Clark, 1986, p. 84), meaning that suggestive information becomes part of memory. Being unable to withstand suggestion implies a risk for erroneous and false testimonies, with potentially far-reaching consequences in a legal process. For instance, Chan et al. (2017) showed that yielding to suggestions in a forensic interview, even when being warned against doing so, altered eyewitness memory in a follow-up interview, making the testimony less accurate.

When individuals are engaged in complex tasks, such as partaking in an investigative interview, workload on cognitive resources is high (Hanway et al., 2021), and such high load has been shown to be related to a higher susceptibility to suggestion (Otgaar et al., 2012). Given that non-native language comprehension and production in itself is cognitively taxing (Green, 1998; Hernandez and Meschyan, 2006; Abutalebi, 2008), it seems possible that investigative interviews with witnesses using a non-native language could result in higher susceptibility to suggestions (Alm et al., 2019). Language barriers between an eyewitness and interviewer can introduce unique challenges, including potential misunderstandings. To navigate these challenges, interviewers often resort to strategies like reformulating and rephrasing questions for clarity. This observation was supported by Allison et al. (2022), who found that interviewers frequently used such strategies with non-native speaking interviewees. While repetition and rephrasing can be part of any interview process, in the context of non-native interviews, there is a potential risk. The interviewee might interpret repeated questions, even if slightly rephrased, as a signal that their initial answer was not satisfactory. This could suggest to them that the interviewer is seeking a different response. This, in turn, might inadvertently lead the interviewer to employ suggestive or leading questions to direct the interviewee toward providing a particular type of response. Even though there is a strong consensus concerning the recommendation to avoid leading questions in police interviews (Lamb et al., 2007), evidence shows that they are commonly posed (Cederborg et al., 2013). If forensic professionals view interviewees who respond in an inconsistent way as less credible, this may introduce more skepticism and suggestions, causing further reductions in the witness’ accuracy. Thus, the link between suggestibility and testimony language seems particularly important to investigate. For this study, while recognizing multiple factors can influence suggestibility, our primary emphasis is on the impact of testifying in one’s non-native language. Given these considerations, we expected that participants testifying in a non-native language will be more susceptible to suggestions than their native speaking counterparts.

### The confidence-accuracy relation and eyewitness language

Forensic investigators almost always obtain statements of confidence from eyewitnesses, and witnesses’ self-reported confidence in their memory often play a crucial role in legal investigations (Yates, 2017; Fitzgerald et al., 2021). Confidence refers to an individual’s belief in their own ability to accurately recall and convey information, reflecting their perception of the correctness of their memory. The relation between eyewitness confidence and accuracy was long debated, but at present there is consensus that eyewitnesses generally express higher confidence in correct than in incorrect details in their eyewitness statements (Juslin et al., 1996; Wixted and Wells, 2017; Lindholm et al., 2018; Gustafsson et al., 2019). Research also shows that accurate memories are expressed with fewer markers of retrieval effort (e.g., delays, non-functional filler words, hedges, and response latency) than inaccurate ones (Lindholm et al., 2018; Gustafsson et al., 2019). Moreover, these effort markers have been found to mediate the relation between witnesses’ judgments of confidence in their memories and their actual accuracy. Hence, witnesses tend to be more confident in the accuracy of memories expressed with less effort than in memories that require effort to retrieve. It is not known however, whether language barriers affect this confidence-accuracy relation. Given that language comprehension and production often are cognitively taxing for non-native speakers (Green, 1998; Hernandez and Meschyan, 2006; Abutalebi, 2008), the effort based on language difficulties may confound the relation between witnesses’ memory accuracy and confidence. Specifically, it seems possible that witnesses testifying in a non-native language may attribute less confidence to their memories regardless of actual accuracy. We, therefore, expected that non-native speakers will generally exhibit lower confidence in their memories compared to native speakers.

### Observers’ judgments of witness credibility and language

The credibility of eyewitnesses has been a subject of extensive research in the field of psychology and criminal justice, and it plays a vital role in the legal process (Wells et al., 2006; Frumkin, 2007; Albright, 2017). Credibility pertains to the extent to which an individual’s testimony or statements are trustworthy and believable (Undeutsch, 1984). If non-native (vs. native) speakers exhibit more effort cues when retrieving their memories, this could potentially affect not only their own confidence judgments but also how observers perceive the quality of the witnesses’ memories. Specifically, if observers use witnesses’ retrieval effort as a cue to memory quality, they may perceive non-native speaking witnesses as less credible than witnesses who report their memory in their native tongue, regardless of witnesses’ actual accuracy. This means that reliable and potentially important information about a criminal case may be dismissed due to a witness’ language difficulties. Accordingly, for a more comprehensive understanding of the role of native vs. non-native language in investigative interviews, we set out to investigate whether independent observers would judge eyewitnesses speaking in a non-native tongue as less credible, compared to their native speaking counterparts.

### The present study

The primary aim of this study was to advance the knowledge on the role of native vs. non-native language in investigative witness interviews, using a design similar to that of Alm et al. (2019). The first focus of the current research was to examine the influence of language on a witness’ memory accuracy and susceptibility to suggestions. Prior research (Alm et al., 2019; Allison et al., 2022) suggests that language barriers may induce greater misunderstandings, potentially engendering suggestions among non-native speakers. Specifically, in Study 1, participants watched a mock-crime film and then gave a video-recorded testimony in either their native (i.e., Swedish) or a non-native (i.e., English) language. We simulated an investigative interview situation by using both free and cued recall. Next, participants were interviewed using the manual for Gudjonsson’s Suggestibility Scales (GSS; Gudjonsson, 1997). Our main hypotheses was that—due to a higher cognitive load—eyewitnesses testifying in a non-native language would (1a) report fewer correct details and (1b) report more incorrect details across free and cued recall, and (2) exhibit a higher degree of suggestibility than native speaking eyewitnesses. We also expected that non-native speaking witnesses would (3) exert a higher degree of self-rated cognitive effort, and (4) expected that they would be judged as less credible by others compared to native speaking eyewitnesses. The second focus delved into the confidence-accuracy relation in testimonies given in a witness’ native or non-native language. Existing research (Lindholm et al., 2018; Gustafsson et al., 2019, 2022) indicates that witnesses’ confidence often hinges on the ease of memory retrieval. Accordingly, we hypothesized that non-native speakers—who may face more challenges in memory retrieval—would exhibit (5a) lower self-rated confidence in reported detail information elicited during cued recall, and (5b) lower self-rated confidence in answers to GSS.^1^ The third focus examined if the language in which a testimony is given affects how others judge the credibility of that testimony, based on the assumption that witnesses providing their testimony in a non-native language would exhibit more effort than non-native speaking witnesses. Specifically, in Study 2, using a subsample of the video-taped testimonies from Study 1, we expected (6) that testimonies from non-native speaking witnesses would be rated as less credible than testimonies from native speaking witnesses. Both studies have been approved by the Swedish Ethical Review Authority (File number 2020-00624).

## Study 1

### Method

Study 1 was preregistered on the Open Science Framework (OSF) prior to data collection.^2^

#### Participants

As the literature on language effects on eyewitness accuracy is scarce, we ran a sensitivity analysis in GPower (Erdfelder et al., 1996), to calculate the minimum detectable effect size. With 60 participants in each condition (in total 120 participants), the study should be sensitive enough to detect an effect size of *d* = 0.51 with power of 80% given α = 0.05, and a two-tailed test.

We recruited a total of 133 participants via an online Swedish research volunteering platform (Accindi.se), the Stockholm University Social Psychology lab’s Facebook group, and through bulletin ads at universities and libraries in the Stockholm area, in exchange for a 50 SEK gift card. As our aim was to test effects of native vs. non-native language on eyewitness testimony, we screened participants’ English language level. Participants were asked to (1) choose the alternative that best suited their English proficiency level (Basic, Independent or Proficient; a shortened version of the Common European Framework of Reference for Languages’ Global Scale, 2019) and (2) rate how comfortable they were speaking in English (1 = not at all comfortable, to 7 = very comfortable). Eight participants who stated that their English proficiency level was “Proficient” in addition to rating that they were very comfortable speaking in English (a 7 on the scale from 1–7) were excluded, as the language manipulation would then have no effect. For seven additional participants who had made the same two ratings we suspected that these ratings did not match the participants’ actual mastery of the English language as evidenced in their video recorded oral testimonies. For this reason, we had an independent rater, blind to the study design and hypothesis, judge their English level, using the same items described above. Since the new ratings were all lower (new ratings can be found at: https://osf.io/xe596/), these seven participants were included. From the remaining sample, four additional participants were excluded due to technical issues (e.g., poor internet connection), leaving a total sample of 121 participants.

In the final sample there were 92 women (76%, *M*_age_ = 30.30, SD_age_ = 9.39), 28 men (23%, *M*_age_ = 32.50, SD_age_ = 10.10), and one person who indicated other gender, ranging in age from 18 to 60 years, with 60 participants (45 women) in the native language group and 61 participants (47 women, 13 men, and one with other gender) in the non-native group. Within the non-native language condition, four participants rated their English proficiency level as Basic, 34 participants as Independent, 22 participants as Proficient. The rating was missing for one participant. The median rating of how comfortable they were speaking in English was 6 (*M*_English_ = 5.30, SD_English_ = 0.91).

#### Materials and procedure

Due to the COVID19-pandemic, the investigative interview was recorded in Zoom whereas all other tasks and ratings were made in the survey tool Qualtrics (i.e., informed consent, viewing the mock-crime video, filler-task, confidence judgments, perceived credibility, perceived cognitive effort, and demographics, respectively). After consenting to participate in the study, participants were randomly allocated to either the native (i.e., Swedish) or non-native (i.e., English) language condition. The interviews were conducted by the same interviewer in both conditions, who, while having proficient English skills and strictly following the interview protocol, also spoke the non-native language in the non-native condition. Participants were informed that they would not be able to pause, replay or zoom in on the mock crime film, and that they were not allowed to take notes. Participants were then presented with a 36-s muted video of a mock crime, filmed from an eyewitness point-of-view (Gustafsson et al., 2021). The film showed an outdoor scene with a man (the victim) approaching and starting a conversation with another man (the first perpetrator) next to a car in an empty parking lot. Next, a third man (the second perpetrator) stepped out of the car and stabbed the victim with a knife while the first perpetrator grabbed a hold of the victim from behind. The perpetrators left the victim badly injured and entered the car where a third person had been sitting in the driver’s seat all along.

After viewing the film, participants completed a filler-task consisting of 10 incomplete black-and-white pictures of different objects and animals which they were to identify and name, with a 2-min time limit. Before the witness interview, participants in the non-native language condition were told that the interview was to be held in English. No instruction about language was given to participants in the native language condition. Participants were then interviewed about their memory of the crime first through free recall, where participants were asked to give an exhaustive and detailed description of the crime as if they had witnessed it in real life. The free recall was followed by a cued recall task in which participants answered seven open-ended questions (e.g., “You mentioned a person who got assaulted, can you describe what that person looked like?”). During the cued recall phase, as the witness reported their testimony, the interviewer wrote down as many of the details as possible reported by the witness (e.g., “red jacket,” “silver car,” “victim shorter than perpetrator”) on a sheet with numbered lines.

Lastly, susceptibility to suggestions was investigated using the Gudjonsson Suggestibility Scales - GSS (without the delay, see Smeets et al., 2009) adapted to the crime scenario used in the present study. The GSS consists of five non-leading “true” questions (e.g., “Did the crime event take place on a parking lot?”) and 15 leading questions (e.g., “Was there blood from the victim on the ground?”), totalling 20 questions. Five of the 15 leading questions included only false options (e.g., “Was the victim stabbed two or three times?,” the correct answer is one time). The questions were posed in the order according to the GSS manual (Gudjonsson, 1997). Once participants had responded to the 20 questions (Yield 1), they received mock negative feedback that some of their answers were incorrect. All 20 questions were then posed and answered again (Yield 2). All three parts of the investigative interview, that is free recall, cued recall and GSS, were recorded on video. Participants were then asked about their confidence, ranging from 0 to 100% with 20% integers, in answers to each Yield 2 question, one by one. It should be noted that confidence refers to an individual’s belief in their ability to recall and communicate information accurately, reflecting the correctness of their memory. Participants next rated their confidence with regard to each specific detail from the cued recall that the interviewer had previously written down.

Then, participants rated their own perceived credibility, which refers to the extent to which they believe their statements or testimonies are likely to be perceived as trustworthy and believable. Finally, participants rated their cognitive effort and provided demographic information, including age, gender, and educational level. Perceived credibility was measured using eight items (e.g., “How accurately did you remember the depicted event?” and “How useful do you think your testimony would be in a crime investigation?”; Lindholm, 2008) on a Likert scale from 1 (not at all) to 7 (very much). In the final analyses, seven of these items were used since one (“How nervous were you during the interview?”) exhibited low factor loading (Cronbach’s α for the final index = 0.78). Self-reported cognitive effort (Englert et al., 2015; Alm et al., 2019) was measured using four items (e.g., “How effortful did you find the task?” and “How mentally exhausted do you feel right now?”) on a Likert scale ranging from 1 (not at all) to 7 (very much). Three items were retained in the cognitive effort index (Cronbach’s α = 0.61) since the fourth item (“How much effort did you have to exert to make sure your story was accurate?”) exhibited low factor loading. Materials and interview protocols are available at: https://osf.io/xe596/.

##### Coding

The videotaped testimonies were transcribed verbatim. The coding template for memory accuracy for the free and cued recall phases cataloged all scorable details based on the content of the film (Gustafsson et al., 2021). For details to be scored as (in)correct, wordings had to match (deviate from) the descriptions in the coding template. We scored each detail in the statements, with each correct detail receiving 1 correct point, and each incorrect detail receiving 1 incorrect point. For example, a statement such as “the victim’s jacket was not black, not blue nor green” (correct answer = red) would receive 3 correct points, and a statement such as “He had a green bag” (where “bag” = correct, and “green” = incorrect) would receive 1 correct and 1 incorrect point. In cases where details were repeated within or across free and cued recall, participants were only given a score the first time they had given that particular information. When participants rated their confidence in details elicited during cued recall (e.g., “he had a green bag”), the interviewer repeated the entire statement and the participant was asked to rate their confidence in the accuracy of that specific statement, in this case, the assertion that the bag was green. The details given during cued recall for which participants gave confidence judgments were coded for accuracy (1 = correct, 0 = incorrect) using the same coding template and protocol described above (*N* = 1,649; *n*_correct_ = 1,135; *n*_incorrect_ = 514), with 829 details in the native language condition (*n*_correct_ = 564; *n*_incorrect_ = 265) and 820 details in the non-native language condition (*n*_correct_ = 571; *n*_incorrect_ = 249). Two raters independently coded the same 10% of the transcribed free and cued recall interviews in order to establish accuracy (correct and incorrect detail information) and one of them coded the remaining 90%. The intra-class correlation coefficient (ICC) was computed to assess the agreement between the two raters; ICC_correct_ = 0.98 (95% CI [0.90, 0.99]), ICC_incorrect_ = 0.87 (95% CI [0.57, 0.96]). Disagreements between the raters were resolved through discussion.

The answers to the GSS, totalling *N* = 4,840, were coded in accordance with the GSS manual (Gudjonsson, 1997). Participants obtained four suggestibility indices: Yield 1, Yield 2, Shift, and Total suggestibility. Yield 1 refers to the scores obtained from giving in to the 15 leading questions in the first round of questioning, ranging from 0 to 15 (Cronbach’s α = 0.80). Yield 2 refers to the scores obtained from giving in to the 15 leading questions after receiving negative feedback, ranging from 0 to 15 (Cronbach’s α = 0.84). Shift score, ranging from 0 to 20, is a measure of a distinct change in the answer in either direction (e.g., from correct “no” to incorrect “yes”; from incorrect “stabbed two times” to correct “no, stabbed once”) in response from Yield 1 to Yield 2 (Cronbach’s α = 0.74). Shift scores also include the five non-leading questions. Total Suggestibility is the sum of Yield 1 and Shift scores, ranging from 0 to 35 (Cronbach’s α = 0.98). For the final GSS analyses, 2,418 Yield 1 statements, 2,386 Yield 2 statements, 2,385 Shift scores, and 2,385 Total Suggestibility scores were usable (36 Yield 1 and Yield 2 statements were excluded due to the interviewer posing the wrong question, misunderstandings of instructions and/or internally inconsistent answers; see Supplementary material for detailed report on data handling).

#### Data analysis

In our preregistration, we decided *a priori* to test hypotheses 2 and 5a using *t*-tests with Holm correction comparing the experimental conditions. However, given the repeated nature of our dependent measure (all participants produced several scores on each GSS measure [hypothesis 2] as well as several confidence judgments [hypothesis 5a]), we conducted multilevel modeling with individual responses nested within participants. All analyses were carried out using R (RStudio Team, 2020; R Core Team, 2022). Multilevel analyses were carried out with *lme4* (Bates et al., 2015), *lmerTest* (Kuznetsova et al., 2017) to use Satterthwaite’s method for approximating degrees of freedom for the t-tests, and *AICcmodavg* (Mazerolle, 2020) for model selection using Akaike weights (see Burnham and Anderson, 2002; Wagenmakers and Farrell, 2004); the Holm correction with *multcomp* (Bretz et al., 2016); the intra-class correlation coefficient using *irr* (Gamer et al., 2012); reliability analyses using *psych* (Revelle, 2022), and data wrangling and visualization were made using *tidyverse* (Wickham et al., 2019). A detailed report of the hypothesis as well as exploratory tests can be found at: https://osf.io/xe596/.

### Results and discussion

First, we examined the contribution of each covariate (i.e., age, gender, educational level) on the outcome variables using hierarchical multiple regression analyses, adding covariates as predictors in a set of models. As no covariate was statistically significant, these analyses are not reported further. Second, we ran a mediation analysis to examine the role of confidence as mediator of the language-memory accuracy relation. As this mediation analysis was not statistically significant, we do not report it further (the code, analyses and output can be found at: https://osf.io/xe596/).

#### Memory accuracy, perceived cognitive effort and self-perceived credibility

Next, we examined the effects of language on accuracy, perceived cognitive effort and perceived credibility. Descriptive and inferential statistics are presented in Table 1. For accuracy, we found that native (vs. non-native) speakers tended to provide both slightly more correct, and also more incorrect, details, although not statistically significantly (see Table 1). When comparing the groups on an accuracy index (total number of incorrect details subtracted from total number of correct details), native (vs. non-native) speakers provided slightly, but not significantly, more accurate testimonies. For self-reported cognitive effort, we found that native (vs. non-native) speakers reported slightly, but not significantly, more cognitive effort. Finally, for perceived credibility, we found that native (vs. non-native) speakers tended to perceive themselves as more credible, but again the results were not statistically significant. As none of these results were statistically significant, we report unadjusted *p*-values instead of the Holm-corrected *p*-values.

**Table 1:** Descriptive and inferential statistics for memory accuracy, self-reported perceived cognitive effort and credibility.

	Native eyewitnesses (*n* = 60)	Non-native eyewitnesses (*n* = 61)	*t*	*p*	Cohen’s *d* [95% CI]
	*M* (SD)	*M* (SD)			
Correct	36.88 (10.12)	35.52 (10.66)	0.72	0.474	0.13 [−2.39, 5.10]
Incorrect	7.62 (3.86)	7.41 (4.31)	0.28	0.782	0.05 [−1.27, 1.68]
Index^a^	29.27 (9.47)	28.11 (10.13)	0.65	0.519	0.12 [−2.38, 4.68]
Cognitive effort	4.68 (1.00)	4.38 (1.07)	1.57	0.118	0.29 [−0.07, 0.67]
Credibility	5.18 (0.76)	5.10 (0.68)	0.61	0.542	0.11 [−0.18, 0.34]

df = 119.

a Accuracy index was computed by subtracting total number of incorrect details from total number of correct details.

#### GSS: Yield 1, Yield 2, Shift, and Total suggestibility for native and non-native witnesses

To test the effect of language on GSS (hypothesis 2), we used multilevel modeling with language (native as reference group, compared to non-native) predicting the different suggestibility variables (Yield 1, Yield 2, Shift and Total suggestibility) as outcomes in separate analyses. In our GSS analyses, we compared baseline null-models with intercept of suggestibility and random intercept for participants only; against models adding language (native vs. non-native) as fixed effects. As GSS scores are binary (1 = yield/shift, 0 = no-yield/no-shift), we performed generalized linear mixed-effects modeling. For Total Suggestibility, we performed a linear mixed-effects model. In sum, we found no statistically significant main effects of language (native vs. non-native) on witnesses’ susceptibility to suggestions with regard to any of the GSS outputs (Yield 1, Yield 2, Shift, and Total suggestibility). The results of GSS predictor models are displayed in Table 2.

**Table 2:** Mixed models predicting GSS from witnesses’ language: Yield 1, Yield 2, Shift and Total Suggestibility (with native language as reference group).

	*b* [SE]	*z*		OR	95% CI	
					LL	UL
**Yield 1**						
Intercept	−2.10 [0.11]	−19.12		0.12	0.10	0.15
Non-native language	−0.01 [0.15]	−0.10		0.98	0.73	1.32
**Yield 2**						
Intercept	−1.88 [0.14]	−13.87		0.15	0.12	0.20
Non-native language	−0.29 [0.19]	−1.51		0.75	0.52	1.09
**Shift**						
Intercept	−2.47 [0.17]	−14.37		0.08	0.06	0.12
Non-native language	−0.33 [0.24]	−1.40		0.72	0.45	1.14
	***b* [95% CI]**	**SE**	***t***	**df**	***p*-value**	
**Total suggestibility**						
Intercept	0.22 [0.18, 0.26]	0.02	10.85	119.92	<0.001	
Non-native language	−0.03 [−0.08, 0.03]	0.03	−0.89	120.23	0.373	

For Yield 1, Yield 2 and Shift: b = logistic coefficients; SE = Standard errors of the logistic coefficient estimates; z = z-value of coefficient; OR = odds ratio; CI = confidence interval; LL = lower limit; UL = upper limit.

For Total Suggestibility: Participants: 120. Number of observations: 2385. *b* = coefficients; CI = confidence interval; SE = Standard errors of the coefficient estimates; t-tests use Satterthwaite approximations for degrees of freedom.

The Yield 1 result showed a non-significant tendency in line with our expectations, where non-native witnesses yielded slightly more to suggestions (*M* = 2.35, SD = 1.79) than native witnesses (*M* = 2.27, SD = 1.52). However, the Yield 2 result was contrary to our expectations since native speaking witnesses yielded slightly more to suggestions (*M* = 3.12, SD = 2.59) than non-native speaking witnesses (*M* = 2.60, SD = 2.31). Also, the Shift result was contrary to our expectations, where native speaking witnesses made slightly more changes to their answers between the first and the second round of questioning after receiving negative feedback (*M* = 2.04, SD = 2.26) than did non-native speaking witnesses (*M* = 1.70, SD = 2.02). Similar unexpected results were found for Total Suggestibility, where native witnesses were slightly more susceptible to suggestions (*M* = 4.31, SD = 2.99) than non-native witnesses (*M* = 4.04, SD = 3.40). For our model comparisons using Akaike weights (values ranging from 0 to 1, where larger values indicates stronger evidence compared to other models being considered), the baseline null-models had best-fit for Yield 1 *w_i_*(AIC) = 0.73, Shift *w_i_*(AIC) = 0.51, and Total Suggestibility *w_i_*(AIC) = 0.65. For Yield 2, the best-fit model included language as fixed effect, *w_i_*(AIC) = 0.53.

#### Confidence-accuracy relation for native and non-native witnesses

Finally, we tested the effect of witnesses’ language on the confidence-accuracy relation (hypothesis 5a) using a linear mixed-effects model with accuracy (correct vs. incorrect) and language (native as reference group, compared to non-native) predicting confidence judgments on individual statements. We compared a baseline null-model with intercept of confidence judgment and random intercept for participants only; against models adding predictors as fixed effects. Results showed that the model including both accuracy, language, and their interaction, had the best fit; *w_i_*(AIC) = 0.97. We found a significant main effect of accuracy, suggesting that overall, witnesses were more confident in correct than incorrect details. There was also a statistically significant main effect of language on confidence, showing that native speaking witnesses were overall more confident (*M* = 87.10, SD = 20.86) than non-native speaking witnesses (*M* = 83.49, SD = 23.01; *d* = 0.16). We found no interaction between accuracy and language. Results of the confidence-accuracy by language model are displayed in Table 3.

**Table 3:** Results from the Linear mixed model predicting confidence by accuracy and language (with native language as reference group).

	*b* [95% CI]	SE	*t*	df	*p*
Intercept	82.47 [79.23, 85.71]	1.66	49.77	302.34	**<0.001**
Accuracy	6.63 [3.59, 9.66]	1.55	4.29	1603.67	**<0.001**
Non-native language	−4.76 [−9.38, −0.14]	2.36	−2.02	314.99	**0.045**
Accuracy × non-native language	1.70 [−2.62, 6.04]	2.21	0.77	1602.34	0.442

Statistically significant *p*-values boldfaced. Number of participants: 121. Number of observations: 1,649. b = coefficients; CI = confidence interval; SE = Standard errors of the coefficient estimates; *t*-tests use Satterthwaite approximations for degrees of freedom.

Taken together, the results largely show similarities across the two language groups (see Tables 1, 2). These results contrast with our hypotheses, as we expected non-native speakers to be less accurate (Hypothesis 1a-b), more susceptible to suggestion (Hypothesis 2; Alm et al., 2019), experience more cognitive effort (Hypothesis 3; Green, 1998; Hernandez and Meschyan, 2006; Abutalebi, 2008) and perceive themselves as less credible (Hypothesis 4). A straightforward explanation is that participants in the non-native group were quite proficient in their non-native language, minimizing potential effects of speaking in a non-native language. This is also supported by the self-rated language proficiency scores, which were fairly high. In turn, this suggests that non-native speakers can be as accurate and reliable as eyewitnesses as native speakers. However, previous studies on the effects of language on memory accuracy show divergent results (Alm et al., 2019; Ernberg and Mac Giolla, 2022; Hu and Naka, 2022) and are also few in number, meaning that there is not conclusive data to support any specific direction of outcome. This highlights the need for more research on the effects of language on memory accuracy. In contrast to the similarities between the two groups, non-native speakers were less confident overall in their memories compared to the native speakers (see Table 3). Given the difference between non-native witnesses’ self-rated credibility, and the confidence they themselves expressed in their memories, the question is how external observers would view them. In our second study, we examined how observers judged credibility in verbal eyewitness testimonies from native and non-native speakers. Given that forensic researchers currently recommend legal staff to rely on witnesses’ confidence judgments (e.g., Mickes et al., 2017; Wixted and Wells, 2017; Wixted et al., 2018, 2021), eyewitness testimonies from non-native speakers might then get a smaller impact in a criminal case, as high confidence supposedly indicates accuracy.

## Study 2

Although participants in Study 1 perceived themselves as being equally credible regardless of language testified in, this might not actually be the case for observers *judging* the eyewitnesses (Lindholm, 2005; Lindholm, 2008). Thus, in Study 2, we examined how observers judged credibility in verbal eyewitness testimonies from native and non-native speakers.

### Method

Study 2 was preregistered on the Open Science Framework (OSF) prior to data collection.^2^

#### Participants

Two-hundred and ninety-three native Swedish-speaking participants (91 excluded; see Supplementary material for exclusion criteria) were recruited from Stockholm University campus, Accindi.se, and the department’s Social Psychology lab Facebook group, to independently judge witness credibility. In the final sample (*N* = 202) there were 147 women (72.77%, *M*_age_ = 32.86, SD_age_ = 12.17), 54 men (26.73%, *M*_age_ = 36.43, SD_age_ = 12.16), and one person who indicated other gender, ranging in age from 18 to 76 years (*M* = 33.94, SD = 12.32, mdn = 30), with 99 participants (67 women) randomly allocated to the native speaking witness language group and 103 participants (80 women, 22 men, and one with other gender) to the non-native speaking witness group. All participants gave written informed consent to participate.

#### Materials and procedure

We first selected the stimulus material by sampling 36 eyewitness testimonies out of the 121 in Study 1 (nine random samples of native speaking women; nine ditto of native-speaking men; nine non-native speaking women; nine non-native speaking men; see Supplementary material for screening process). Using the survey tool Qualtrics, participants in Study 2 were randomly assigned to view either a native or non-native speaking eyewitness. After consenting to participate in the study, participants received instructions that they were to watch an investigative interview with a person who had witnessed a serious violent crime and that they were to imagine themselves in the role of an interrogator. Participants were randomly allocated to one of the 36 videotaped testimonies, and then rated eyewitness credibility. Credibility ratings were made along the same eight items as described in Study 1 (Lindholm, 2008; Cronbach’s α = 0.83).^4^

#### Data analysis

As in Study 1, all analyses were carried out using R (Rstudio, Team, 2020; R Core Team, 2022). Multilevel analyses were carried out with *lme4* (Bates et al., 2015), *lmerTest* (Kuznetsova et al., 2017) to use Satterthwaite’s method for approximating degrees of freedom for the t-tests, and *AICcmodavg* (Mazerolle, 2020) for model selection using Akaike weights (see Burnham and Anderson, 2002; Wagenmakers and Farrell, 2004).

### Results and discussion

To examine the effects of language on observer-judged eyewitness credibility, we used multilevel modeling. We also examined potential effects of witness gender. Thus, we compared a baseline null-model with intercept of credibility judgment and random intercept for witness only against two models that added language and witness gender, respectively, as fixed effects. Using Akaike weights for model comparison, the model including only language had best fit; *w_i_*(AIC) = 0.49. We found a statistically significant main effect of language (Estimate = −0.36, SE = 0.18, df = 33.55, *t* = −2.05, *p* = 0.047), in which witnesses in the non-native condition were judged as less credible (*M* = 4.51, SD = 1.04) compared to those in the native condition (*M* = 4.85, SD = 0.80; *d* = 0.37). We found no main effect of witness gender on credibility (Estimate = 0.15, SE = 0.18, df = 33.55, *t* = 0.82, *p* = 0.419). The random effects showed that the credibility scores varied significantly between witnesses (SD = 0.39).

In sum, the results from Study 2 revealed that non-native witnesses were perceived as less credible than their native counterparts by independent judges, supporting our hypothesis (6). Thus, even though both native-speaking and non-native speaking witnesses had similar recall accuracy, as shown in Study 1, non-native speaking witnesses were nevertheless judged as less credible.

## General discussion

The primary aim of Studies 1 and 2 was to advance our knowledge regarding the role of native vs. non-native language in investigative witness interviews. First, we examined the effects of language on a witness’ memory accuracy and susceptibility to suggestion. Contrasting previous findings (Alm et al., 2019), we found no statistically significant difference in memory accuracy or suggestibility between native and non-native speakers (see Tables 1, 2). Second, we examined the confidence-accuracy relation in testimonies given in a witness’s native or non-native language. In line with extant research (Juslin et al., 1996; Wixted and Wells, 2017; Lindholm et al., 2018; Gustafsson et al., 2019), our findings showed higher confidence for correctly recalled details compared to incorrectly recalled details’ (see Table 3). Moreover, non-native speakers were less confident in their recall compared to native speakers (see Table 3), providing empirical support for the notion that non-native speakers likely encounter more challenges in memory retrieval. Third, we examined whether the language in which a testimony was given affected how others judged the credibility of that testimony. Our results effectively supported the hypothesis that testimonies from non-native (vs. native) witnesses would be judged as less credible. Overall, these findings provide valuable insights into the role of language in eyewitness testimonies, although further investigation is warranted.

A major take-home message from the present study is the further expansion of the confidence-accuracy relation—with confidence pertaining to an individual’s belief in their ability to recall and communicate information accurately. Previous research has shown that people are generally more confident in their own correct than incorrect statements (Sporer et al., 1995; Wixted and Wells, 2017; Gustafsson et al., 2019). This was also the case in our data across language conditions. However, despite the fact that native and non-native speaking participants displayed negligible differences in memory accuracy, non-native participants were generally less confident in their memories. As confidence judgments are related to accuracy (Juslin et al., 1996; Wixted and Wells, 2017; Lindholm et al., 2018; Gustafsson et al., 2019), this finding may have practical relevance for legal practitioners as they often acquire statements of confidence from interviewees (Yates, 2017; Fitzgerald et al., 2021). Thus, if legal practitioners rely on witness confidence, this could result in systematic biases against testimonies provided by non-native interviewees. Future research should scrutinize legal practitioners’ perspectives and basis for reliability judgments on testimonies where there are language barriers present.

### Credibility judgments

In our data, the difference between native and non-native participants’ self-rated perceived credibility, that is the extent to which they believe their statements or testimonies are likely to be perceived as trustworthy and believable, was not statistically significant. However, the forensic relevance of self-rated credibility judgment is debatable. More importantly, although we found negligible differences in memory accuracy between native and non-native speaking eyewitnesses in Study 1, the non-native speaking eyewitnesses were judged as less credible than their native-speaking counterparts by observers in Study 2. This finding has practical implications, as it highlights potential biases in testimony evaluation that may significantly impact the fairness of legal proceedings. Also, non-native speaking interviewees often belong to an ethnic outgroup, which can engender increased skepticism regarding their testimonies (Scassa, 1994; Lindholm, 2005; Frumkin, 2007; Lindholm and Yourstone Cederwall, 2013). It is therefore crucial to recognize that when language difficulties intersect with foreign background or ethnicity, the situation becomes even more intricate, especially if these intertwined factors contribute to attributions of lower credibility. That independent judges rate non-native speakers as less credible could lead to unjust outcomes in cases involving non-native witnesses. However, it is important to acknowledge that the independent judges in the current study were laypeople without training, as opposed to legal professionals. Also, one potential confounder to consider is that the raters in the non-native condition were actually assessing statements that were non-native to them, which could inherently influence their judgments.

### Susceptibility for suggestions

Expanding upon the Alm et al. (2019) study, which only measured Yield 1 and had a smaller sample size, the current study carried out the complete GSS procedure. Our findings revealed similar trends for Yield 1, suggesting that non-native (vs. native) speaking participants were slightly more susceptible to suggestions in the initial round of questioning. However, this difference was somewhat small and not statistically significant. For Yield 2, Shift, and Total Suggestibility, our data showed the opposite results, suggesting that native speaking participants were slightly more susceptible to suggestions than non-native speaking participant. Again however, these effects were not statistically significant. These findings, which indicated that native speakers were more susceptible to suggestions and experienced higher cognitive effort, were counterintuitive compared to our initial reasoning but actually align with the findings by Otgaar et al. (2012), wherein participants yielded more to suggestions when experiencing a higher cognitive effort. While the differences observed were negligible, their potential implications in real-world settings, without real-world interview nuances like rephrasing or repetition, cannot be underestimated. Misinformation from even a single round of questioning can jeopardize the reliability of testimony, particularly in high-stakes situations. Future research should investigate this language-suggestibility relation further in order to clarify these findings.

### Limitations

There are several methodological limitations that need to be considered. First, in Sweden, people are generally proficient in English and, in the present study, most of our non-native participants were moderately to highly proficient in the English language. Needless to say, to improve generalizability, an ideal setting would have been to compare native speaking eyewitnesses against low proficient non-native speaking eyewitnesses. However, observed differences between these groups, such as lower confidence for non-native speakers, and being judged as less credible, should likely be exacerbated in non-native samples with less ability in the non-native language. Thus, our findings call for conceptual replications with eyewitnesses testifying in languages at lower proficiency levels. Second, our design, wherein both the interviewer and participants communicated in a non-native language in the non-native condition, introduces potential confounds. Although having a consistent interviewer across conditions minimized variability, it might have influenced participants to use simpler language, possibly affecting their confidence in their responses. While the interviewer demonstrated proficiency and strictly followed the interview protocol, this design choice complicates distinguishing between the effects of the participant’s and the interviewer’s language proficiency. Future research should carefully consider this aspect to ensure clarity of results. Third, due to the pandemic, our witness interviews were performed digitally via Zoom. As a consequence, most participants conducted the experiment in a familiar, non-stressful physical environment. Although we believe that this setting is largely equivalent to testing participants in a laboratory (participants provided a similar amount of details and accuracy rates as in the laboratory studies by Gustafsson et al., 2019 and Lindholm et al., 2018), it is unclear how and to what extent this might have affected the outcome, and especially the effects of the simulated investigative interview setting. Fourth, for our self-rated cognitive effort manipulation check, we used the original items validated within the field of sport psychology, as outlined by Englert and Bertrams (2014) and Englert et al. (2015). It is possible that this measure does not readily translate to a manipulation check in a legal setting. Fifth, concerning the ecological validity, as with most studies within the eyewitness research paradigm (see Albright, 2017), these findings do not necessarily generalize to real-world cases. In contrast to real-world eyewitnesses, our participants were informed prior to the experiment that they were going to see a mock crime event and then answer questions about the event. Nonetheless, this is a typical design used by researchers as the best proxy. Also, this study is hitherto the largest conducted between-subject design investigating the effects of native vs. non-native language on eyewitness testimonies and do provide the research field with informative data.

### Conclusion

In conclusion, our study did not show effects of language on witnesses’ memory accuracy, susceptibility to suggestions, or self-rated cognitive effort. However, our results corroborated the confidence-accuracy relation, showing that both native and non-native speaking witnesses were more confident in correct than incorrect details. Crucially, we found that non-native speaking witnesses were less confident in their memories, even if their memory performance was similar to native-speaking witnesses. Furthermore, in Study 2, independent judges perceived non-native speaking witnesses as less credible compared to native speakers, emphasizing the significant role that language plays in evaluating credibility. These findings have practical implications for the legal system, highlighting the importance of training and awareness among legal professionals and jurors to recognize and address biases due to language barriers—ensuring just and objective assessment of all testimonies. Future research should investigate other aspects of the role of language in eyewitness reports, such as legal practitioners’ experiences and perceptions of language barriers when conducting interviews with non-native interviewees. Additionally, it would be valuable to explore specific subgroups of non-native eyewitnesses, such as those with a low vs. high proficiency in the non-native language.

## Data availability statement

The datasets presented in this study can be found in online repositories. The names of the repository/repositories and accession number(s) can be found at: https://osf.io/xe596/.

## Ethics statement

The studies involving humans were approved by Swedish Ethical Review Authority (File number 2020-00624). The studies were conducted in accordance with the local legislation and institutional requirements. The participants provided their written informed consent to participate in this study.

## Author contributions

AR: writing—original draft, writing—review and editing, conceptualization, methodology, formal analysis, investigation, data curation, and visualization. TL: writing—review and editing, conceptualization, methodology, resources, visualization, supervision, project administration, and funding acquisition. PG: writing—review and editing, resources. CA: writing—review and editing, conceptualization, methodology, resources, visualization, supervision, project administration, and funding acquisition. All authors contributed to the article and approved the submitted version.

## Conflict of interest

The authors declare that the research was conducted in the absence of any commercial or financial relationships that could be construed as a potential conflict of interest.

## Publisher’s note

All claims expressed in this article are solely those of the authors and do not necessarily represent those of their affiliated organizations, or those of the publisher, the editors and the reviewers. Any product that may be evaluated in this article, or claim that may be made by its manufacturer, is not guaranteed or endorsed by the publisher.

## Footnotes

^1^This preregistered hypothesis was not tested. A detailed explanation can be found in the Supplementary material.

^2^https://osf.io/zywuk

^4^Before the credibility ratings, participants were first instructed to formulate four questions for an upcoming follow-up interview, with the goal of finding out information that could help solving the crime. After generating interview questions, participants rated the credibility of the eyewitness. Both the interrogation questions and the ratings of credibility are part of a different preregistered study, and only the credibility ratings will be reported in the present study.
