# Supplementary material for: Memory accuracy, suggestibility and credibility in investigative interviews with native and non-native eyewitnesses
Source: Front Psychol. 2023 Aug 24;14:1240822. doi: 10.3389/fpsyg.2023.1240822 (PMC10484215; doi:10.3389/fpsyg.2023.1240822)
Supplement: Supplementary file 1 [file Data_Sheet_1.docx]

**Memory accuracy, suggestibility and credibility in investigative interviews with native and non-native eyewitnesses**

Arman Raver, Torun Lindholm, Philip U. Gustafsson and Charlotte Alm

**Supplementary Materials**

Supplementary Study 1 - section 1

*Coding and scoring GSS*. The answers to the GSS, totalling N = 4840, were coded in accordance with the GSS manual (Gudjonsson, 1997). Participants obtained four suggestibility indices: Yield 1, Yield 2, Shift, and Total Suggestibility. Yield 1 refers to the scores obtained from giving in to the 15 leading questions in the first round of questioning, ranging from 0 to 15 (Cronbach's α = 0.80). Yield 2 refers to the scores obtained from giving in to the 15 leading questions after receiving negative feedback, ranging from 0 to 15 (Cronbach's α = 0.84). Shift score, ranging from 0 to 20, is a measure of a distinct change in the answer in either direction (e.g., from correct “no” to incorrect “yes”; from incorrect “stabbed two times” to correct “no, stabbed once”) in response from Yield 1 to Yield 2 (Cronbach's α = 0.74). Shift scores also include the five non-leading questions. Total Suggestibility is the sum of Yield 1 and Shift scores, ranging from 0 to 35 (Cronbach's α = 0.98). For the final GSS analyses, 2418 Yield 1 statements, 2386 Yield 2 statements, 2385 Shift scores, and 2385 Total Suggestibility scores were usable.

When scoring suggestibility, the GSS manual states that, in some cases, subjects can give internally inconsistent answers (e.g., “Yes, I’m not sure, probably not”) which should be addressed by requesting clarification (e.g., “You have given me more than one answer; which is the one you are most happy with?”; Gudjonsson, 1997, p. 12). However, in our data, answers to the GSS questioning were not always clear-cut (i.e., “yes”, “no”, or “don’t know” answers). Rather, participants would often deliberate their responses back-and-forth out loud (e.g., *“… Two or three? I think he was only stabbed once. That I could see. I mean they were really close together, so he might have stabbed him multiple times, but I could only see the knife entering him once*.” or “… *eh I can’t remember that I sa, eh saw that. But I was like eh very focused on the.. the stabbing so it might have happened that ehm yeah might have happened I don’t know*”).

In total, 132 Yield 1 and Yield 2 statements included internally inconsistent answers of which 17 were resolved by the interviewer requesting a clarification as the GSS manual states (Gudjonsson, 1997). However, in the remaining 115 statements, the interviewer forgot to request clarifications, or the interviewer did not request clarifications due to, with simultaneous interpretation, erroneously deeming that theses answers were satisfying (i.e., yielding or non-yielding). As a solution, instead of excluding this data, when coding for suggestibility, the authors resolved this issue by implementing a principle stating that the final self-correction or change in answer in a statement is definitive (viz. yielding or non-yielding). Accordingly, in the example above “… *Two or three? I think he was only stabbed once. That I could see. I mean they were really close together, so he might have stabbed him multiple times, but I could only see the knife entering him once.*”, the participant did not give in to the leading question “*Was the victim stabbed 2 or 3 times with a knife?*” as the concluding “*I could only see the knife entering him once*.” was deemed as definitive. To illustrate with another example, the answer “.. *oh that is a very hard question. I can’t really say that. He was eh hit. I saw the that eh he was hit in the stomach. B .. but I can’t say that he was hit .. eh kicked .. by any of them. But eh anything is possible*” to the leading question “*Did the perpetrator / any of the perpetrators kick the victim when he had fallen to the ground?*”, did in fact yield a suggestibility score.

In total, 64 statements in Yield 1 with internal inconsistencies were not confronted in line with the GSS manual, resulting in 16 changes in scoring in accordance with the instated principle. In Yield 2, 51 statements with internal inconsistencies were not confronted in line with the GSS manual, resulting in 13 changes in scoring in accordance with the instated principle. As a result, a total of 27 changes in shift scores were made following the implementation of the principle. For the final GSS analyses, the corrected scoring was used.

Supplementary Study 1 - section 2

*Deviations from preregistration*. We intended to obtain self-rated confidence on GSS Yield 2 answers (hypothesis 3a). However, and related to the abovementioned issue with the GSS in section 1, answers were not always clear-cut when coding the GSS-confidence data for correctness. When the interviewer simultaneously wrote down Yield 2 answers on a sheet with numbered lines corresponding to the GSS question during the investigative interview, the interviewer’s notes did not always correspond to the participant’s statement (e.g., the answer *“.. nno I didn’t see that. But it’s possible that he could have a scarf*” was noted as “*no didnt see that*” to the GSS question “*Did the victim have a scarf?*”). Consequently, as data was contaminated erroneously, the self-reported confidence in GSS Yield 2 answers were excluded in the final analyses. Thus, hypothesis 3a was not possible to investigate and was therefore omitted.

Supplementary Study 2 - section 1

Of the 121 video-taped eyewitness recordings, we selected a subsample of 36 videotaped eyewitness testimonies (nine native speaking women; nine native speaking men; nine non-native speaking women; nine non-native speaking men; see supplemental material for rigorous screening process). We conducted a rigorous screening where we excluded (1) testimonies with poor audio and/or video quality, (2) bad internet connection, (3) testimonies with contaminated data (e.g., with misunderstanding, participants with statements such as “the camera panned away”), (4) disturbances in setting (e.g., unusual camera angels, noise and movements in the periphery), and (5) non-native participants with high English proficiency (so that the language manipulation had an effect). Then, of the remaining testimonies we conducted a rigorous screening process where we selected those testimonies with matching (a) length of testimony, (b) audio and/or video noise, and (c) performance relative to memory accuracy, self-reported perceived cognitive effort and perceived credibility. For this part, we only used the free recall and added a new audio intro by the interviewer using the software DaVinci Resolve. Descriptive and inferential statistics of the 36 eyewitnesses can be found here: <https://osf.io/vyz96>.

Supplementary Study 2 - section 2

In part 2, we recruited an additional 293 participants of which 91 were excluded. First, we screened participants’ English language using one item “*How well would you say that you understand spoken English?*” on a 1 (Not at all) to 7 (Very well) Likert scale. Twelve participants rating their English level ≤ 2 was excluded. Two participants did not consent and hence excluded. Furthermore, ten participants were excluded as they did not pose any usable questions for an upcoming planned study, and 14 participants were excluded as they did not adhere to the instructions. Finally, participants entering the survey more than once (n = 51) were excluded.
